# Can we tackle the inverse care law in general practice?

**DOI:** 10.3399/BJGP.2025.0363

**Published:** 2025-07-22

**Authors:** David N Blane, Carey Lunan, Stewart W Mercer

**Affiliations:** 1 Senior Clinical Lecturer, School of Health & Wellbeing, University of Glasgow, Glasgow, UK; 2 Honorary Clinical Senior Lecturer, College of Medicine and Veterinary Medicine, University of Edinburgh, Edinburgh, UK; 3 Professor of Primary Care and Multimorbidity, Centre for Population Health Sciences, Usher Institute, University of Edinburgh, Edinburgh, UK

The inverse care law (ICL), as described by Julian Tudor Hart in his seminal 1971 *Lancet* article, is the observation that *‘The availability of good medical care tends to vary inversely with the need for it in the population served.’* ^1^ Tudor Hart’s main target was the threat of marketisation in health care, made clear in the second (less quoted) part of the ICL: *‘This inverse care law operates more completely where medical care is most exposed to market forces, and less so where such exposure is reduced.’*

Whether or not you agree with Tudor Hart’s thesis on market forces, it is clear that the distribution of general practice resources is not targeted to areas of greatest need. A 2022 report from The Health Foundation found that there were fewer GPs, despite higher health needs, in the most socioeconomically disadvantaged areas of England.^2^ We conducted a similar analysis of the general practice workforce in Scotland and found that, not only were there fewer GPs in the most deprived areas, but there were also fewer non-GP clinical staff (for example, practice nurses) and fewer non-clinical staff.^3^

In our systematic scoping review, we found that, since Scottish devolution in 1999, a range of policies and interventions have sought to address health inequalities in general practice, with varying success and sustainability;^4^ only 2 of the 20 interventions — community links workers and financial advisers — have been rolled out nationally, though sustainability is uncertain. In this editorial, we discuss a range of developments to improve the volume and quality of general practice in the most socioeconomically deprived areas, and what more can be done.

## Greater investment in general practice everywhere

The first step to address the ICL is greater overall investment in general practice. Despite close to 90% of NHS encounters taking place in primary care, the proportion of spending on general practice across the UK nations sits at <7% of the overall NHS budget and has been declining^3,5^. This is despite general practice being *‘one of the most fiscally responsible parts of our health system … represent*[ing] *one of the greatest returns on investment.’* ^5^ The Royal College of General Practitioners (RCGP), British Medical Association (BMA), and several politicians have called for this share of funding to increase, up to 15%. It would be politically unpalatable, and destabilising for general practice more widely, to redistribute existing scarce resources to deprived areas without this additional investment across the board first being secured, but this should be supported by improved financial transparency and governance arrangements.

## Proportionate universalism

In May 2024, the RCGP report *Breaking the Inverse Care Law in UK General Practice* made recommendations under five overarching domains to foster more equitable and inclusive health care in general practice (Table 1).^6^ Among these was the Marmot concept of ‘proportionate universalism’ — that to *‘reduce the steepness of the social gradient in health, actions must be universal, but with a scale and intensity that is proportionate to the level of disadvantage.’* ^7^ Public Health Scotland have recently published guidance on proportionate universalism^8^ and the new Enhanced Service for cardiovascular disease in Scotland is a practical demonstration of how it might be implemented.^9^

**Box 1.** RCGP recommendations for addressing the inverse care law

Domain	Selected recommendations
Access and navigation to healthcare services	Policymakers, commissioners, and providers of health services across the UK should offer tailored support for socioeconomically deprived, underserved, and inclusion health groups, as part of efforts to improve patient access.
Equitable distribution of resources	All general practice funding streams should be reviewed to better match resources with needs, in alignment with the principles of proportionate universalism, alongside increased investment across general practice.
Workforce enhancement	Governments should evaluate existing GP recruitment and retention schemes across the UK to ensure they focus on supporting recruitment and retention in socioeconomically deprived areas and implement additional schemes where needed.
Utilisation of data	Ensure all practices across the UK have access to high-quality data and analytical tools that facilitate understanding of their community’s health needs.
Impact of social determinants of health	Governments across the UK should produce a cross-government strategy to reduce health inequalities, which recognises and commits to reducing the impact of social determinants on population health, using every available policy lever and is underpinned by the necessary funding.

## Supporting the workforce

It is no surprise that, in more socioeconomically deprived (and typically under-doctored) areas, where patient needs and complexity are high, GPs report higher stress and patients report lower enablement and satisfaction compared with more affluent areas.^2,10^ To avoid a self-perpetuating cycle of recruitment and retention difficulties, we need a broad range of supports for the primary care workforce (including the wider multidisciplinary team [MDT]) targeting different career stages.^11^

In March 2023, the Scottish Government developed the Inclusion Health Action in General Practice programme, funding general practices in the most socioeconomically deprived areas in Glasgow to improve community connections, enhance workforce knowledge and skills, and provide outreach and extended consultations.^12^ This initiative incorporates many of the elements that were common among the more effective interventions in our scoping review, including increased clinician time to engage with complex clinical work; use of outreach and system flexibility to better engage with those at risk of ‘missingness’,^13^ coordinating community engagement, bridging between health care and third sector; and effective MDT working and staff buy-in.^4^ However, it is recognised that this programme is only an interim solution to increase funding to support general practice to address health inequalities,^14^ a ‘proof of concept’ of what could be achieved with more equitable and adequate resource. The recent Audit Scotland report on general practice performance following the implementation of the 2018 General Medical Services (GMS) contract, highlighted that the Scottish Government’s efforts to support general practices to address health inequalities have had limited impact,^15^ despite this being a stated aim of the 2018 GMS contract. It makes a specific recommendation that the Scottish Government needs to set out a clear plan for how it intends to better support general practices to contribute to tackling health inequalities.^15^

## Making better use of data

There is clear scope in UK general practice for improving systems for collecting and analysing detailed sociodemographic data to identify health inequities. Furthermore, evidence generation to date has been limited by an abundance of small-scale interventions or pilot projects, with short timescales and non-recurring funding, making it difficult to demonstrate impact or generalisability. Further research is needed to develop and test tools and interventions to improve our response to socioeconomic disadvantage, with rigorous evaluation to assess implementation and effect.

Ultimately, to tackle the ICL in general practice, we need greater overall investment in general practice, with that additional resource allocated at a scale and intensity proportionate to local need (‘proportionate universalism’). This will help create the conditions for services to be more inclusive, intersectional, flexible, community-oriented, and connected across sectors.^16^
